# Association between lipid profile in early pregnancy and the risk of congenital heart disease in offspring: a prospective cohort study

**DOI:** 10.1038/s41598-024-53876-6

**Published:** 2024-02-13

**Authors:** Minli Zhao, Danwei Zhang, Xinrui Wang, Haibo Li, Bin Sun, Zhengqin Wu, Yibing Zhu, Hua Cao

**Affiliations:** 1 College of Clinical Medicine for Obstetrics and Gynecology and Pediatrics, Fujian Medical University, No.18 Daoshan Road, Fuzhou, 350000 China; 2 Department of Cardiac Surgery, Fujian Children’s Hospital (Fujian Branch of Shanghai Children’s Medical Center), College of Clinical Medicine for Obstetrics & Gynecology and Pediatrics, Fujian Medical University, Fuzhou, 350014 China; 3 Fujian Maternity and Child Health Hospital, College of Clinical Medicine for Obstetrics & Gynecology and Pediatrics, Fujian Medical University, Fuzhou, 350000 China; 4 NHC Key Laboratory of Technical Evaluation of Fertility Regulation for Non-Human Primate (Fujian Maternity and Child Health Hospital), Fujian Medical University, Fuzhou, 350014 China

**Keywords:** Lipid profile, Early pregnancy, Congenital heart disease, Offspring, Endocrine system and metabolic diseases, Metabolic disorders, Disease prevention, Paediatrics, Public health, Health care, Medical research, Risk factors

## Abstract

This study aimed to investigate the association of lipid profile in early pregnancy and the risk of congenital heart disease (CHD) in offspring. This study was a prospective cohort design based on the Fujian Birth Cohort Study in China. We recruited pregnant women at ≤ 14 weeks of gestation between 2019 and 2022, and all participants in this study filled out the questionnaire about periconceptional exposure. Simultaneously, we collected participants’ fasting blood samples to measure their lipid profile by automatic biochemical analyzer. The outcome was defined as offspring with CHD. A multivariable logistic regression model was used to calculate adjusted odds ratio (AOR) risk estimates, which indicate the associations between maternal lipid profiles and CHD in offspring. Restricted cubic splines were used to estimate their nonlinear relationship. A total of 21,425 pregnant women with an average gestational age of 11.3 (± 1.40) weeks were included in the analysis. The higher triglyceride (AOR 1.201, 95% CI [1.036, 1.394]), low-density lipoprotein (AOR 1.216, 95% CI [1.048, 1.410]), apolipoprotein B (Apo B) (AOR 2.107, 95% CI [1.179, 3.763]) levels were correlated with increased odds of CHD in offspring, while high-density lipoprotein (OR 0.672, 95% CI [0.490, 0.920]) related with decreased odds of CHD in offspring. The restricted cubic spline suggested a nonlinear relationship between total cholesterol (TC) levels and the risk of CHD in offspring (*P* = 0.0048), but no significant nonlinear relationships were found in other lipid profile. Apolipoprotein A was not related to the risk of CHD in offspring as either a continuous variable or a hierarchical variable. Elevated lipid profile in early pregnancy levels are associated with an increased risk of CHD in offspring. Additionally, there is a non-linear relationship between TC levels and the risk of CHD in offspring.

## Introduction

Congenital heart disease (CHD) is one of the most prevalent congenital malformations, impacting approximately 5–15 per 1000 live births worldwide^1–4^. Although the mortality of CHD has decreased for the advances in diagnostic and therapeutic techniques, its impact on the quality of life can extend to adulthood or even longer^5,6^. Therefore, there is an urgent need to prevent CHD by investigating modifiable risk factors and proposing practical preventive strategies.

The embryo is susceptible to the influence of maternal metabolic factors during pregnancy. Maternal lipid profile, as one of these crucial prenatal metabolic factors, plays a significant role in the occurrence and progression of congenital malformations^7^ such as neural tube defects^8^, autism spectrum disorder^9^. However, limited but controversial studies have investigated the association between lipid profile levels during pregnancy and the risk of CHD in offspring. A previous case–control study conducted in Rotterdam reported that mildly deranged lipid profile, determined at around 16 months after the index pregnancy, were associated with an increased the risk of CHD in offspring^10^. However, the critical period for cardiac tissue differentiation and development occurs in early pregnancy^11^. Another prospective cohort study in Amsterdam explored the association of maternal triglyceride (TG) levels during early pregnancy with congenital anomalies in offspring, and the subgroup analysis for specific congenital anomalies showed that the U-shaped association was most prominent for cardiovascular congenital anomalies^7^. However, the study didn't show the adjusted results for cardiovascular congenital anomalies. Furthermore, a retrospective nested case–control study from Shanghai also showed a positive association between lipid profile levels and the risk of CHD in offspring^12^. However, the researchers did not do the linear or nonlinear relationship between lipid profile and the risk of CHD in offspring. So far, few studies with sufficient sample sizes have investigated verified the association between lipid profile in early pregnancy and the risk of CHD in offspring, and this important issue remains unclear.

This study, based on a large birth cohort in southeast China, aims to investigate the association between lipid profile levels in early pregnancy and the risk of CHD in offspring, including exploring the nonlinear relationship between them.

## Methods

### Study design and population

**Figure 1:**
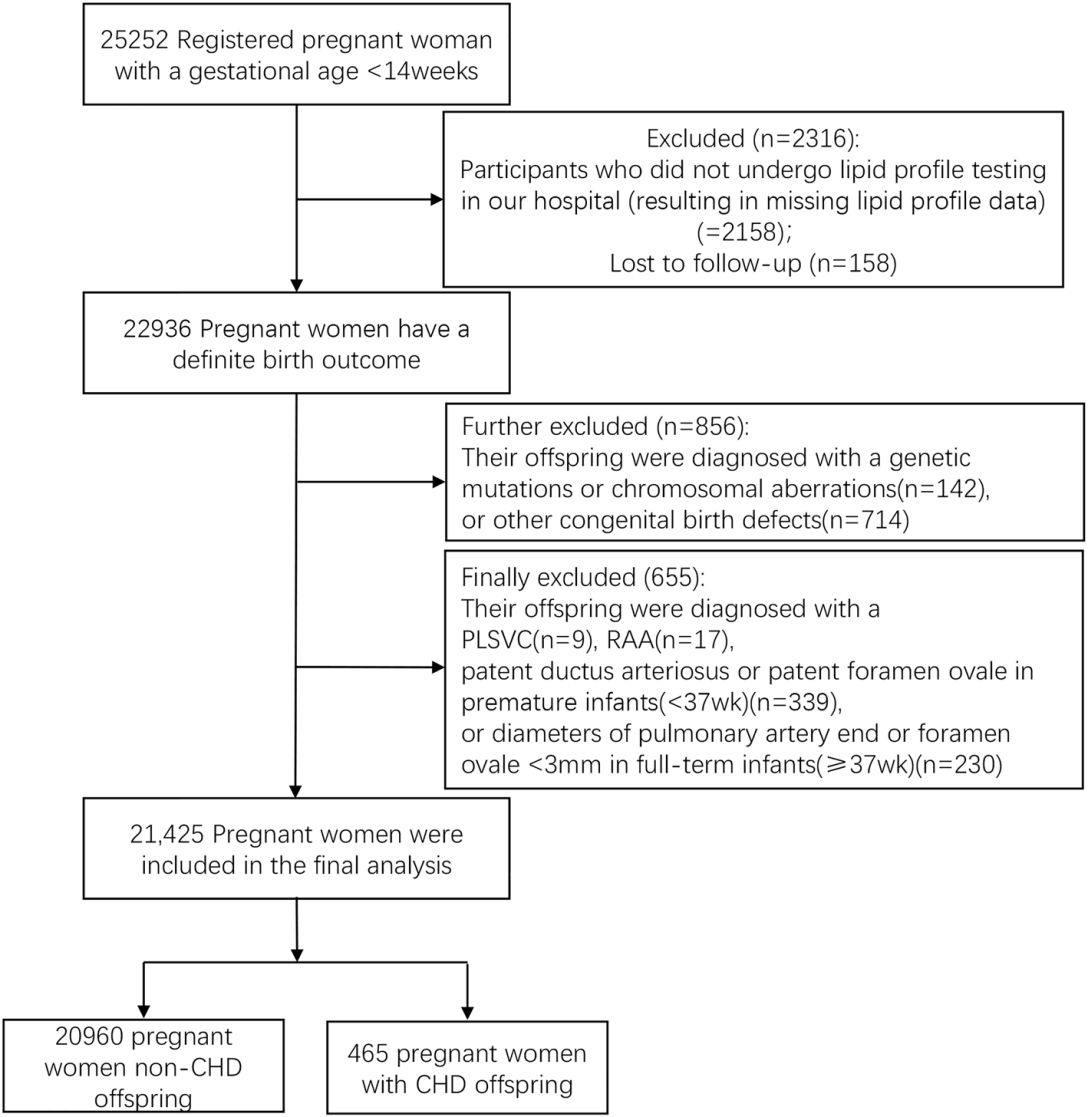
Flow chart of the study cohort and identification of fetus with congenital heart disease. *PLSVC* persistent left superior vena cava, *RAA* right aortic arch, *CHD* congenital heart disease.

This prospective population-based cohort study was conducted based on the Fujian Birth Cohort Study (FJBCS), which was commenced in 2019 at Fujian Maternal and Child Health Hospital in Fujian, China for exploring the relationship between perinatal exposure and adverse pregnancy outcomes. The method and design details of the FJBCS study have been previously described^13^. Briefly, pregnant women of gestational age ≤ 14 weeks were invited to complete the registration during their first prenatal visit at our hospital. Gestational age is calculated using the date of the last menstrual period, and ultrasound is used to calculate gestational age only when the menstruation is irregular. Pregnant women diagnosed with multiple pregnancies, severe cardiovascular disease, cerebrovascular disease, liver or kidney failure, or family history of CHD were not enrolled in the study. This study recruited a total of 25,252 participants who completed early pregnancy examinations between January 2019 and February 2022. All participants received face-to-face interviews conducted by trained nurses and filled out a questionnaire. Simultaneously, we collected their fasting blood samples. Among these participants, we first excluded those who did not undergo lipid profile testing in our hospital (resulting in missing lipid profile data) and those who were lost to follow-up. We further excluded pregnant women whose offspring had genetic mutations, chromosomal aberrations, or other congenital birth defects. Finally, we excluded pregnant women whose offspring were diagnosed with clinically nonsignificant forms or spontaneously resolved defects, including persistent left superior vena cava (PLSVC), right aortic arch (RAA), patent foramen ovale or patent ductus arteriosus in premature infants (< 37 weeks), or the diameters of patent foramen or pulmonary artery end < 3 mm in full-term infants (≥ 37 weeks)^12,14–16^. The final analysis included 21,245 singleton pregnant women and their fetuses. Figure 1 showed the participant flowchart. All procedures of this study were approved by the ethics committee of Fujian Provincial Maternal and Child Health Hospital (Approval number: 2017KR-030) and written informed consent was signed by every participant. We also confirmed that all research was performed in accordance with Declaration of Helsinki.

### Lipid profile and other biochemical indicators

At the early pregnancy prenatal visit (about 11 weeks gestation), lipid profile including total cholesterol (TC), TG, high-density lipoprotein (HDL), low-density lipoprotein (LDL), apolipoprotein A (Apo A), and apolipoprotein B (Apo B), as well as other biochemical indicators such as fasting blood glucose, alanine aminotransferase (ALT), aspartate aminotransferase (AST), free thyroxine (FT4), and thyroid stimulating hormone (TSH) were tested after a fasting period of more than 8 h using latex-enhanced immunoassay and enzymatic procedures on an automatic biochemical analyzer. The process of biochemical analyses was performed in the following steps: (1) Peripheral blood was collected during the early pregnancy prenatal visit. (2) The whole blood was collected into a coagulation tube and transferred to the laboratory within 10 min. After incubating at room temperature for 20 min, the tubes were centrifuged at 3000 rpm for 20 min. The supernatant was carefully collected as a plasma sample. (3) Biochemical indexes were analyzed using corresponding kits on an automatic biochemical analyzer (ABBOTT, ACCELERATOR, a3600, USA).

### Outcome

The outcome was defined as offspring with or without CHD. Each case of CHD was diagnosed according to the standardized criteria outlined in the internationally recognized framework known as the International Classification of Diseases version 10 (ICD-10), and subtypes were classified by the codes Q20–Q26. Cases of CHD include live births, stillbirths, and aborted fetuses diagnosed with CHD. Fetuses underwent comprehensive screening for potential congenital defects using basic ultrasound imaging techniques. Echocardiography was employed to meticulously screen fetuses that showed suspicion of having a congenital heart defect. The confirmation of all CHD cases was achieved through a collective effort involving highly skilled professionals, including ultrasound doctors, obstetricians, pediatric cardiac surgeons, and pediatric physicians. For combined cardiac defects, the primary diagnosis was determined based on either the defect requiring the earliest intervention or the most hemodynamically significant structural anomaly.

### Other covariates

In this study, the confounders were collected from self-reported questionnaires (Table S1) or physical examination^13,17^ and defined as follows: (1) Sociodemographic factors: maternal age, ethnicity (Han and ethnic minorities), residence (urban and rural), educational level (12, 12–16 and > 16 years), and monthly income in CNY (4500, 4500–9000 and > 9000 yuan); (2) Obstetric characteristics of this pregnancy: gravidity (0, 1, ≥ 2), assisted reproduction; (3) Health status: body mass index (BMI) and BMI categories (underweight, normal weight, overweight, and obesity), hypertension, medication history (any medication other than non-nutritive substances); (4) Environmental pollution: exposure to air pollution; (5) Lifestyle characteristics: smoking, passive smoking, alcohol consumption; (6) Nutritional supplements: vitamin supplementation, folic acid supplementation, DHA supplementation, cod-liver oil supplementation, and calcium supplementation. For environmental pollution, lifestyle characteristics, and nutritional supplements, the time window of exposure is from the preconception period (within 3 months before conception) to the first trimester of pregnancy (conception to 12 weeks). Except for those specifically pointed out, all the other confounders above were binary variables as yes or no. BMI was calculated by dividing weight (kg) by the square of height (m^2^). The height and weight of pregnant women were measured during registration in early pregnancy, and the pre-pregnancy/early pregnancy BMI was obtained since weight gain is limited during this stage^18^. The BMI category was determined based on the Chinese adult BMI classification standard as follows: underweight (< 18.5 kg/m^2^), normal weight (18.5–< 24 kg/m^2^), overweight (24–< 28 kg/m^2^), and obesity (≥ 28 kg/m^2^)^19^. The blood pressure was measured while sitting using an upper arm oscilloscope, and the average value was obtained by taking at least 3 readings. Hypertension was defined as a blood pressure measurement of ≥ 140/90 mmHg^20^.

### Statistical analysis

For baseline characteristics, continuous variables are expressed as mean ± standard deviation, while categorical variables are presented as number (percentages). The proportion of missing variables ranged from 0.05 to 0.3%. As a result, the continuous variable (BMI) was replaced with the mean value, while the categorical variables (Monthly income and Hypertension) were filled with the mode, representing the highest proportion. Descriptive analysis for continuous variables was conducted using Student's t-tests, and chi-square tests or Fisher's exact tests were performed for categorical variables. Multivariable logistic regression models were used to calculate adjusted odds ratios (AORs) and their 95% confidence intervals (CIs) while controlling for covariates. Covariates were selected based on the results of univariate analysis, taking into account clinical significance, literature evidence, and the causal inference framework established by a directed acyclic graph (DAG) (Fig. S1). Additionally, a collinearity test was conducted to assess the interrelationship between variables. The variables we finally included and specific steps are as follows: (1) Model 1, adjusted for gravidity, assisted reproduction, medication history, exposure to air pollution, and passive smoking. (2) Model 2, adjusted for variables in Model 1 as well as fasting blood glucose and BMI. The concentrations of lipid profiles were divided into quartiles, with the 25th to 75th percentile serving as the reference group. We used restricted cubic spline curves based on logistic regression models with four knots at the 5th, 35th, 65th, and 95th percentiles to assess the nonlinear relationship between levels of lipid profiles and the odds ratios of CHD.

#### Subgroup analysis

Because BMI was demonstrated to impact on CHD in offspring, multivariable logistic regression models were used to explore the relationship between lipid profile in different BMI categories and the risk of CHD in offspring.

#### Sensitivity analysis

To avoid the influence of extreme values, we excluded pregnant women with lipid profile below the 2.5th percentile and above the 97.5th percentile, and then re-evaluated the association between these lipid profile and the risk of CHD in offspring using multivariable logistic regression models.

All statistical analyses were performed using SPSS software version 27 and R software version 4.2.3. A *P* value of less than 0.05 was considered statistically significant.

## Results

### Population characteristics

From January 2019 to February 2022, a total of 21,245 pregnant women were included in our final study, with a mean (± SD) gestational age of 11.3 (± 1.40) weeks. In this study, 465 women delivered CHD offspring, while 20,960 women delivered non-CHD offspring, with the CHD rate of 2.17%. The baseline characteristics of pregnant women are presented in Table 1. Compared with other participants in the study, pregnant women with higher BMI levels (*P* < 0.001), primiparity (*P* = 0.003), assisted reproduction (*P* = 0.016), medication history (*P* = 0.043), exposure to air pollution (*P* = 0.002), and passive smoking (*P* = 0.018) were more likely to give birth to CHD offspring.

**Table 1:** Baseline characteristics of mothers with or without CHD offspring in early pregnancy.

Characteristic	Total (n = 21,425)	CHD (n = 465)	No CHD (n = 20,960)	*P*
Age^†^	31.88 (± 4.06)	31.94 (± 4.097)	31.88 (± 4.060)	0.620
BMI (kg/m^2^)^‡^				0.000
< 18.5	3074 (14.3)	57 (12.30)	3017 (14.10)	
18.5–< 24	14,671 (68.5)	303 (65.20)	14,368 (67.10)	
24–< 28	2940 (13.7)	73 (15.70)	2867 (13.40)	
≥ 28	740 (3.5)	32 (6.90)	708 (3.30)	
Ethnicity (han)^‡^	20,976 (97.9)	9 (1.9)	440 (2.1)	0.807
Residence (urban)^‡^	19,449 (90.8)	421 (90.5)	19,028 (90.8)	0.857
Education level (years)^‡^				0.382
< 12	4842 (22.6)	116 (24.9)	4728 (22.6)	
12–16	15,085 (70.4)	321 (69.0)	14,764 (70.4)	
> 16	1498 (7.0)	28 (6.0)	1470 (7.0)	
Monthly income, CNY^‡^				0.143
< 4500	9177 (42.9)	215 (46.2)	8962 (42.9)	
4500–9000	8735 (40.8)	187 (40.2)	8548 (39.9)	
> 9000	3513 (16.4)	63 (13.5)	3450 (16.5)	
Gravidity^‡^				0.003
0	7897 (36.9)	179 (38.5)	7718 (36.8)	
1	1476 (6.9)	49 (10.5)	1427 (6.8)	
≥ 2	12,052 (56.3)	237 (51.0)	11,815 (56.4)	
Assisted reproduction^‡^	1747 (8.2)	52 (11.2)	1695 (8.1)	0.016
Hypertension^‡^	336 (1.6)	9 (1.9)	327 (1.6)	0.379
Medication history^‡^	10,387 (48.5)	247 (53.1)	10,140 (48.4)	0.043
Exposure to air pollution^‡^	9617 (44.9)	242 (52.0)	9375 (44.7)	0.002
Smoking^‡^	478 (2.2)	6 (1.3)	472 (2.3)	0.165
Passive smoking^‡^	7106 (33.2)	178 (38.3)	6928 (33.1)	0.018
Alcohol consumption^‡^	2497 (11.7)	45 (9.7)	2452 (11.7)	0.179
Vitamin supplementation^‡^	11,229 (52.4)	243 (52.3)	10,986 (52.4)	0.947
Folic acid supplementation^‡^	13,106 (61.2)	280 (60.2)	12,826 (61.2)	0.669
DHA supplementation^‡^	1679 (7.8)	41 (8.8)	1638 (7.8)	0.426
Cod-liver oil supplementation^‡^	246 (1.1)	6 (1.3)	240 (1.1)	0.771
Calcium supplementation^‡^	2499 (11.7)	58 (12.5)	2441 (11.6)	0.583

*CHD* congenital heart disease, *BMI* body mass index.

^†^Mean (SD), Student’s t-test.

^‡^n (%), chi-square test.

### Serum lipid profile

The serum lipid profile of pregnant women with or without CHD offspring are shown in Table 2. Maternal levels of TG (*P* = 0.002), LDL (*P* = 0.001), and Apo B (*P* = 0.001) were higher among mothers delivering CHD offspring compared to those delivering non-CHD offspring. However, maternal levels of HDL (*P* = 0.001) and Apo A (*P* = 0.016) were lower in pregnant women delivering CHD offspring compared to pregnant women delivering non-CHD offspring.

**Table 2:** Blood biochemical indexes of mothers with or without CHD offspring in early pregnancy.

Characteristics	CHD (Mean ± SD)	Non-CHD (Mean ± SD)	*P*
n	465	20,960	–
Blood glucose (mmol/L)	4.79 ± 0.70	4.75 ± 0.42	0.203
TC (mmol/L)	4.70 ± 0.79	4.63 ± 0.74	0.090
TG (mmol/L)	1.45 ± 0.57	1.37 ± 0.53	0.002
HDL (mmol/L)	1.63 ± 0.29	1.68 ± 0.30	0.001
LDL (mmol/L)	2.50 ± 0.65	2.40 ± 0.40	0.001
Apo A (g/L)	1.46 ± 0.28	1.49 ± 0.27	0.016
Apo B (g/L)	0.75 ± 0.17	0.73 ± 0.16	0.001
ALT (U/L)	17.36 ± 13.90	17.05 ± 16.39	0.681
AST (U/L)	17.65 ± 6.25	17.62 ± 8.21	0.994
FT4 (ng/dL)	1.33 ± 0.22	1.34 ± 0.27	0.252
TSH (uIU/mL)	1.11 ± 0.94	1.13 ± 1.05	0.566

*SD* standard deviation, *CHD* congenital heart disease, *TC* total cholesterol, *TG* triglyceride, *HDL* high-density lipoprotein, *LDL* low-density lipoprotein, *Apo A* apolipoprotein A, *Apo B* apolipoprotein B, *ALT* alanine aminotransferase, *AST* aspartate aminotransferase, *FT4* free thyroxine, *TSH* thyroid stimulating hormone.

### Relationships between lipid profile in early pregnancy and CHD in offspring

**Figure 2:**
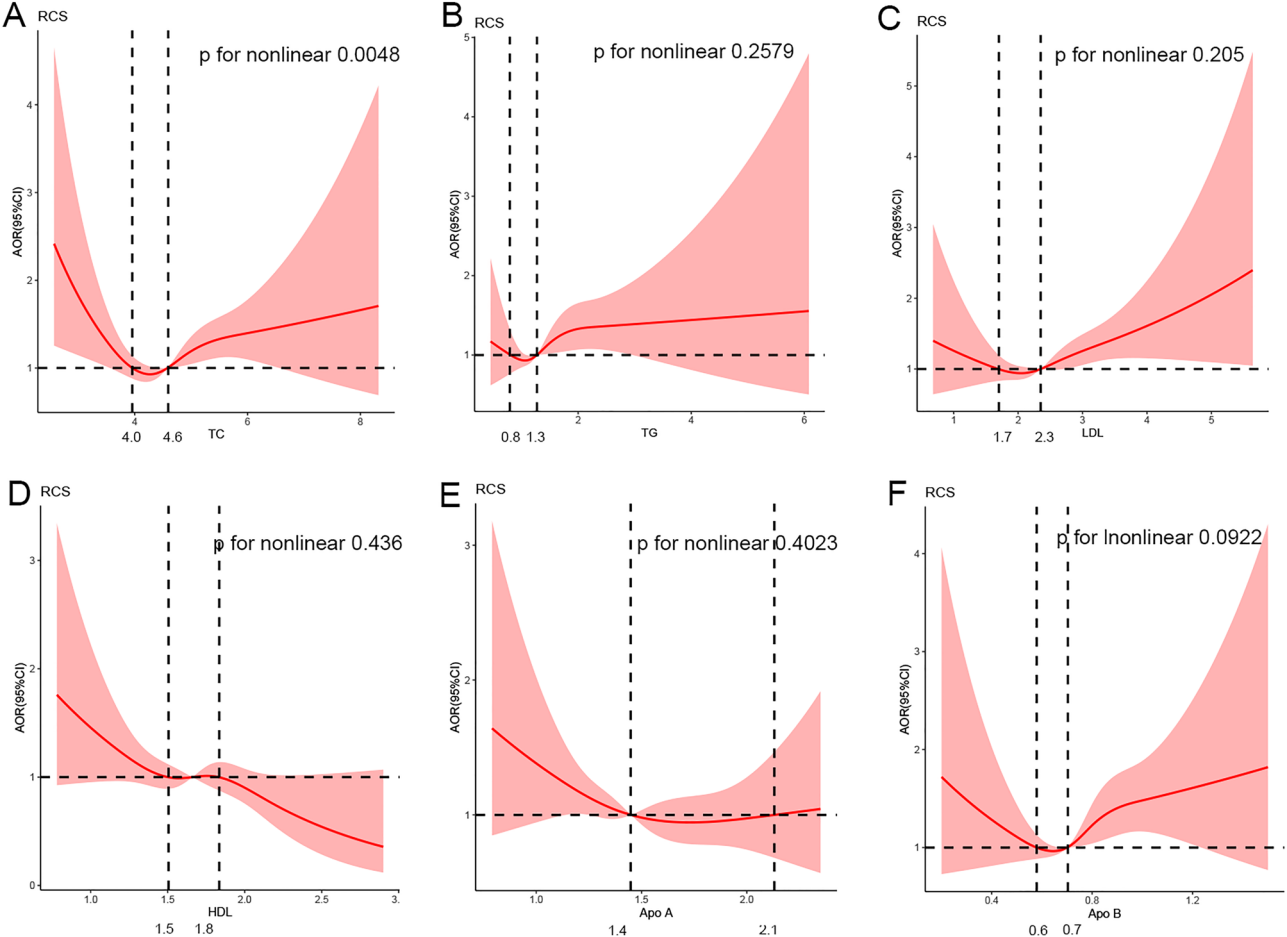
Non-linear relationship between lipid profile in early pregnancy and risk of CHD in offspring. Adjusted odd rates for CHD risk in offspring according to levels of lipid profiles on a continuous scale. Deep red lines are multivariable adjusted odd ratios, with light red areas showing 95% confidence intervals derived from restricted red area regressions with four knots (5th, 35th, 65th and 95th percentiles). The reference for no association is indicated by the solid horizontal dotted lines at an odds ratio of 1.0, and the vertical dotted line indicates the intersection point of the mean lipid curve with the OR reference line of 1.0, the lipid range above the horizontal lines indicates the higher risks of CHD in offspring. The analyses were adjusted for gravidity, assisted reproduction, medication history, exposure to air pollution, passive smoking, blood glucose, and BMI. Table S2 presented the sample size for the CHD and non-CHD cases at different percentiles.

As shown in Table 3, after adjusting for all the confounders, compared to the reference group (25th–75th percentile, 4.14–5.08), pregnant women with TC levels in early pregnancy above the 75th percentile (AOR 1.452, 95% CI [1.166, 1.808]) significantly increased the risk of CHD in offspring. In addition, pregnant women with some lipid profile above the 75th percentile, including TG (AOR 1.249, 95% CI [1.003, 1.555]), LDL (AOR 1.420, 95% CI [1.144, 1.764]), and Apo B (AOR 1.430, 95% CI [1.139, 1.795]) had a 24.9%, 42.0%, and 43.0% higher risk of CHD in offspring, respectively, when compared to the reference group (TG: 1.03–1.59, LDL: 1.99–2.75, Apo B: 0.6–0.8). When the lipid profile as continuous variables, elevated TG (AOR 1.201, 95% CI [1.036, 1.394]), LDL (AOR 1.216, 95% CI [1.048, 1.410]) and Apo B (AOR 2.107, 95% CI [1.179, 3.763]) levels were positively correlated with the risk of CHD in offspring after adjusting for the covariates. Conversely, HDL (AOR 0.672, 95% CI [0.490, 0.920]) was associated with a decreased risk of CHD in offspring. As for the nonlinear relationship shown in Fig. 2 there was a U-shaped nonlinear relationship between TC levels and the risk of CHD in offspring (*P* for nonlinear 0.0048), but no significant nonlinear relationships were found in other lipid profile. Apo A was not related to the risk of CHD in offspring as either a continuous variable or a hierarchical variable.

**Table 3:** Associations between lipid profile in early pregnancy as continuous and categorical variables in early pregnancy and risk of CHD in offspring.

	Unadjusted	Model1	Model2
	OR [95% CI]* P*	AOR [95% CI] *P*	AOR [95% CI] *P*
TC			
Continuous variables	1.120 (0.982, 1.265) 0.068	1.116 (0.987, 1.261) 0.080	1.090 (0.963, 1.233) 0.173
≤ 25th	1.264 (1.008, 1.585) 0.043	1.273 (1.015, 1.597) 0.037	1.297 (1.033, 1.627) 0.025
25th–75th	Reference	Reference	Reference
> 75th	1.488 (1.197, 1.851) 0.000	1.486 (1.194, 1.849) 0.000	1.452 (1.166, 1.808) 0.001
TG			
Continuous variables	1.270 (1.108, 1.455) 0.001	1.265 (1.099, 1.455) 0.001	1.201 (1.036, 1.394) 0.016
≤ 25th	0.899 (0.709, 1.140) 0.378	0.896 (0.706, 1.137) 0.366	0.912 (0.718, 1.158) 0.449
25th–75th	Reference	Reference	Reference
> 75th	1.330 (1.075, 1.645) 0.009	1.333 (1.075, 1.653) 0.009	1.249 (1.003, 1.555) 0.047
HDL			
Continuous variables	0.580 (0.425, 0.791) 0.001	0.608 (0.445, 0.630) 0.002	0.672 (0.490, 0.920) 0.013
≤ 25th	1.293 (1.049, 1.595) 0.016	1.263 (1.024, 1.558) 0.029	1.203 (0.972, 1.487) 0.089
25th–75th	Reference	Reference	Reference
> 75th	0.824 (0.644, 1.053) 0.122	0.832 (0.651, 1.065) 0.144	0.852 (0.666, 1.090) 0.203
LDL			
Continuous variables	1.296 (1.124, 1.485) 0.000	1.274 (1.103, 1.472) 0.001	1.216 (1.048, 1.410) 0.010
≤ 25th	1.069 (0.845, 1.351) 0.579	1.080 (0.854, 1.365) 0.519	1.111 (0.878, 1.406) 0.382
25th–75th	Reference	Reference	Reference
> 75th	1.519 (1.228, 1.880) 0.000	1.489 (1.202, 1.844) 0.000	1.420 (1.144, 1.764) 0.001
Apo A			
Continuous variables	0.652 (0.461, 0.924) 0.016	0.742 (0.520, 1.058) 0.099	0.776 (0.545, 1.106) 0.160
≤ 25th	1.245 (1.008, 1.536) 0.042	1.211 (0.980, 1.496) 0.076	1.171 (0.946, 1.448) 0.147
25th–75th	Reference	Reference	Reference
> 75th	0.777 (0.606, 0.995) 0.046	0.823 (0.641, 1.056) 0.126	0.824 (0.642, 1.058) 0.129
Apo B			
Continuous variables	2.553 (1.460, 4.463) 0.001	2.575 (1.464, 4.528) 0.001	2.107 (1.179, 3.763) 0.012
≤ 25th	0.977 (0.783, 1.219) 0.836	0.980 (0.785, 1.223) 0.857	1.011 (1.139, 1.795) 0.921
25th–75th	Reference	Reference	Reference
> 75th	1.502 (1.201, 1.879) 0.000	1.504 (1.201, 1.883) 0.000	1.430 (1.139, 1.795) 0.002

Cut-off value: TC, 25th: 4.14, 75th: 5.08; TG, 25th: 1.03, 75th: 1.59; HDL, 25th: 1.47, 75th: 1.87; LDL, 25th: 1.99, 75th: 2.75; Apo A, 25th: 1.29, 75th: 1.64; Apo B, 25th: 0.6, 75th: 0.8. Model 1: adjusted for gravidity, assisted reproduction, medication history, exposure to air pollution, passive smoking; Model 2: adjusted for model 1 + blood glucose + BMI.

*CHD* congenital heart disease, *AOR* adjusted odds ratio, *CI* confidence interval, *TC* total cholesterol, *TG* triglyceride, *HDL* high-density lipoprotein, *LDL* low-density lipoprotein, *Apo A* apolipoprotein A, *Apo B* apolipoprotein B.

### Subgroup analysis

**Figure 3:**
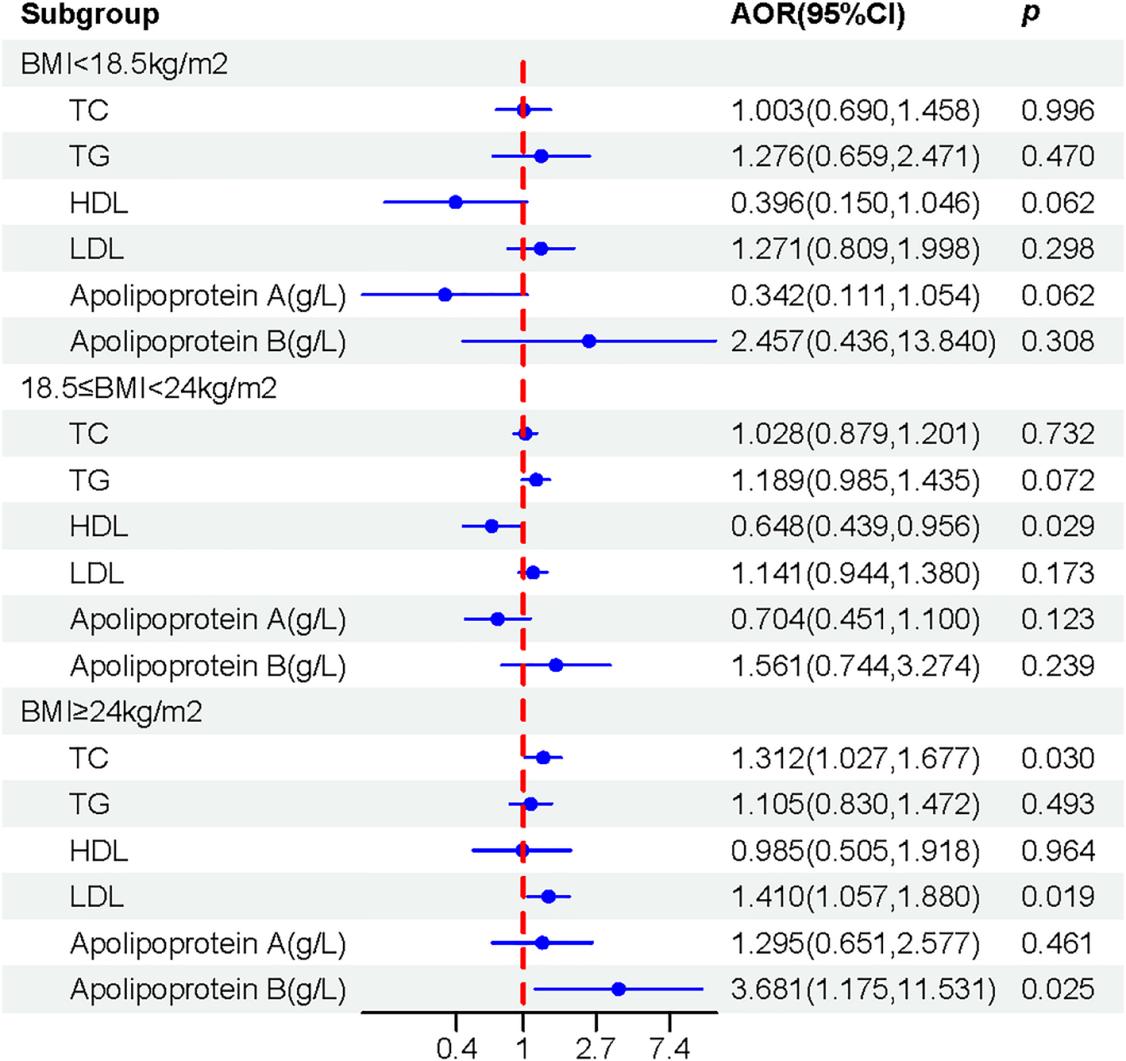
Forest plots showing the relationship between lipid profile in early pregnancy and CHD in offspring stratified by different BMI levels. *TC* total cholesterol, *TG* triglyceride, *HDL* high-density lipoprotein, *LDL* low-density lipoprotein, *BMI* body mass index, *IQR* inter-quartile range, *AOR* adjusted odds ratio, *CI* confidence interval. The analyses were adjusted for gravidity, assisted reproduction, medication history, exposure to air pollution, passive smoking, fasting blood glucose, BMI.

Figure 3 illustrated the relationship between lipid profile in early pregnancy and CHD in offspring, stratified by different BMI levels. After adjusting for potential confounders, we observed that a lipid profile with high TC levels (AOR 1.31, 95% CI [1.03, 1.68]), LDL levels (AOR 1.41, 95% CI [1.06, 1.88]), and Apo B levels (AOR 3.68, 95% CI [1.18, 11.53]) in early pregnancy was associated with an increased risk of CHD in offspring only among overweight/obese pregnant women (BMI ≥ 24 kg/m^2^).

### Sensitivity analysis

After excluding pregnant women with lipid profile levels below the 2.5th percentile and above the 97.5th percentile, the results regarding the relationship between TG, HDL, LDL, Apo B, and CHD in offspring remained robust, as shown in Table S3. The AOR and 95% CIs were as follows: TG (AOR 1.332, 95% CI [1.051, 1.688]), HDL (AOR 0.688, 95% CI [0.475, 0.996]), LDL (AOR 1.326, 95% CI [1.098, 1.601]), and Apo B (AOR 2.109, 95% CI [1.017, 4.371]).

## Discussion

In this prospective birth cohort study, we observed a strong association between elevated lipid profile levels in early pregnancy and increased risk of CHD in offspring after adjusting for covariates, except for HDL and Apo A. When exploring the nonlinear relationship, we discovered a visually significant U-shaped nonlinear relationship between TC and the risk of CHD in offspring, but no significant nonlinear relationships were found in other lipid profile. Maternal lipid metabolism during normal pregnancy can be divided into anabolic and catabolic phases^21^. The first trimester represents a significant anabolic phase^22^. During this period, lipid profile levels continue to increase to meet the growing physiological requirements of the fetus^7,23,24^. However, it has been demonstrated that higher concentrations of lipid profile can increase the risk of birth defects in offspring^7^.

The relationship between lipid profile in early pregnancy and the risk of CHD in offspring remains controversial in previous studies. TC and TG were the two most common lipids in the human body. Smedts et al. first found that maternal elevated TC and TG levels were not associated with the risk of CHD in offspring^10^. After that, Nederlof et al. found that both decreased and elevated TG levels in early pregnancy were associated with increased congenital anomalies risk, especially cardiovascular congenital anomalies, in offspring. Recently, a nested case–control study from Shanghai also found that maternal TG levels in early pregnancy were positively correlated with the risk of CHD in offspring, but TC was not associated with it^12^. Our research has made some additions to the existing studies. For TC, our finding is in accordance with previous study, in which each unit increase in TC levels was not associated with CHD risk. However, different from previous studies, we further found that extremely low and high TC levels (< 25th and > 75th percentile) in early pregnancy were associated with higher CHD risk in the offspring. In addition, we first proved that there was a nonlinear relationship between TC and CHD risk in offspring which prior studies did not take into account. Our result for TG is consistent with the findings of Cao et al.'s study^12^, but differs from the one reported by Smedts et al.^10^ The main reason for the inconsistent results may be attributed to the fact that in Smedts' study, collected the mother's blood samples 16 months after the index pregnancy, however, it is important to note that the critical period of heart development occurs during early pregnancy^11^. Furthermore, their analyses included both fasting and non-fasting cases and control mothers, which may also account for the disparate results^25^.

For other lipid profile, consistent with previous study^10^, our study demonstrated that increased LDL and Apo B levels during pregnancy were associated with an increased risk of having a child with CHD, while Apo A were not. In addition, we first discovered a negative correlation between HDL and the risk of CHD in offspring. High levels of maternal LDL can enter the fetal circulatory system through endothelial cells of the placental barrier^26^, which may potentially affecting the fetal heart development. Conversely, elevated HDL levels may protect the fetal heart from damage. Apo A and Apo B are two proteins related to lipid metabolism: Apo A is involved in the reverse transport and clearance of cholesterol, and it has a protective effect on cardiovascular health^27^; Apo B mediates the transport of cholesterol to tissue cells, and a high level of Apo B is associated with an increased risk of cardiovascular disease^28^. However, we also found other study indicating opposite conclusions as Apo A increased CHD risk, whereas Apo B did not^12^. We speculate that the differences in study population, sample size, and research methods are the reasons why their results differ from ours. Therefore, we recommend conducting further studies to verify this controversial result.

Up to now, the causes of CHD remain mainly unclear, but increasing the environmental factors apart from genetic background^29^. In our study, during the variable selection process, we found that early-pregnancy BMI^30^, gravidity, assisted reproduction^31^, medication history^32^, passive smoking^33^, exposure to air environmental pollution^34^ were associated with CHD risk in offspring, which were also proved to play important roles in CHD development in prior study. Thus, we adjusted those factors to explore the independent impact of lipid profile on offspring CHD in early pregnancy. The conclusion that maternal overweight/obesity increases the risk of CHD in offspring has been confirmed. Overweight/obesity was also associated with a complex set of endocrinologic and metabolic changes, such as directly leading to a significant increase in lipid profile or indirectly affecting blood lipid profile through the development of insulin resistance^35^. Therefore, for the first time, we explored the relationship between lipid profile in early pregnancy stratified by different BMI levels and the risk of CHD. We found that the lack of association between TG, LDL, and Apo B levels and CHD risk in normal and underweight mothers indicates that, in addition to lipid regulation, other crucial steps in lipid metabolism processes may also be affected by maternal overweight/obesity. However, the specific mechanisms of this association need to be further explored through more studies.

Maternal lipid profiles during pregnancy have long been considered a physiological event with little clinical relevance. As a result, the measurement of maternal blood lipid levels is not routinely conducted in most countries, and the corresponding reference ranges remain unclear. However, our results suggest that lipid profiles may lead to adverse outcomes. Although the mechanism of the adverse effects of elevated maternal lipid profile levels on embryonic heart development is not completely clear, it is biologically reasonable. Higher lipid levels trigger chronic inflammation and oxidative stress and produce large amounts of inflammatory factors and free radicals^36^, which have adverse effects on embryonic heart development and angiogenesis^37^. In addition, According to the genetic background of the embryo, exposure to maternal high lipid profile concentration may affect the neural crest, which is involved in embryonic heart development^8^. It is also rational to consider that there exists a non-linear relationship between maternal cholesterol levels and the risk of offspring CHD. Cholesterol plays a crucial role as a major nutrient for fetal growth, serving as a precursor to synthetic steroid hormones and being essential for the development and maturation of fetal organs^38^. Extremely low TC levels can result in birth defects as they fail to meet the nutrient requirements of the developing fetus^39^. However, excessively high TC levels can accumulate in the fetus through the placental barrier, resulting in endothelial dysfunction, increased proinflammatory eicosanoids, and activation of inflammatory pathways^40,41^. Consequently, it has a direct impact on embryonic development and interferes with the normal differentiation of the fetal heart and the development of blood vessels.

The findings of this study have important clinical and public health significance. This study can provide our reference for the determination of lipid profiles in southeast China. Screening lipid profiles during pre-pregnancy and early pregnancy, and providing timely intervention and guidance to women with dyslipidemia can help reduce the incidence of CHD in offspring. In addition, for obese/overweight women of childbearing age, weight control should be timely and strengthen the monitoring of profile lipids.

The major strengths of this study were as follows: First, when compared with previous studies^7,10,12^, this study was conducted as a prospective cohort study with a substantial sample size, which greatly enhances the reliability of the obtained results. Second, six important blood lipid profiles were measured and analyzed during early pregnancy, which was a critical period for cardiac development^16^. Third, this study was the first to demonstrate a nonlinear relationship between TC levels in early pregnancy and the risk of CHD in offspring. However, this study also had several limitations. First, despite adjusting for the majority of important possible covariates, there were still residual covariates. Second, the majority of factors measured in this study were dichotomous variables rather than continuous variables, which had the potential to mask their true associations with CHD. Third, we excluded a subset of the study population for specific reasons. Although the comparison of the characteristics of the two populations suggests only small differences (Table S4), there may still be some selection bias. Therefore, more large-scale studies are needed to address this issue. Fourth, it was important to note that this study is a single-center cohort study conducted in China, which might restrict the generalizability of our findings to other ethnic groups.

## Conclusions

In this study, we discovered that higher TG, LDL, and Apo B levels in early pregnancy were associated with an increased risk of CHD in offspring. Conversely, HDL showed a negative correlation with the risk of CHD in offspring. It is important to consider the potential nonlinear relationship when exploring the influence of lipid profile on CHD risk. Additionally, both serum lipid profile and BMI levels in early pregnancy should be taken into account when assessing their roles in offspring CHD risk. The result of this study indicates that early detection and intervention for lipid profile disorder in early pregnancy may help to reduce the risk of CHD.

### Supplementary Information

Supplementary Information.

## Data Availability

The datasets generated and analyzed during the current study are available from the corresponding author on reasonable request.
