# Supplementary material for: Association between lipid profile in early pregnancy and the risk of congenital heart disease in offspring: a prospective cohort study
Source: Sci Rep. 2024 Feb 13;14:3655. doi: 10.1038/s41598-024-53876-6 (PMC10864369; doi:10.1038/s41598-024-53876-6)
Supplement: Supplementary file 1 — Supplementary Information. [file 41598_2024_53876_MOESM1_ESM.docx]

Table S1. Pregnant women health questionnaire

| 1. Please provide your date of birth: | □□□□ year, □□ month |
| --- | --- |
| 2. Your weight and height before pregnancy: | □□□. □ kg, □□□ cm. |
| 3. What is your nationality? | ① Han ② ethnic minorities _______ |
| 4. Which area have you mainly been living from the preconception period (within 3 months before conception) to the first trimester of pregnancy (conception to 12 weeks)? | ① Urban ② Rural |
| 5. Please tick your education years: | ① <12 ② 12-16 ③ >16 |
| 6. What is your average monthly income? | 1. 0 2. <1500 yuan/month 3. 1501~4500 yuan/month 4. 4501~9000 yuan/month 5. 9001~15000 yuan/month 6. 15001~35000 yuan/month 7. 35001~50000 yuan/month 8. ≥50000yuan/month |
| 7. Have you been pregnant before? | ① No ② Yes, this is the □□th pregnancy |
| 8. What was your method of conception this time? | ① Natural conception  ② Assisted reproduction |
| 9. Please list the names, usage and duration of all drugs used. (except for trace element preparations, vitamins and other nutrients) from the preconception period (within 3 months before conception) to the first trimester of pregnancy (conception to 12 weeks)? | ① No ② Yes |
| 10. Are you frequently exposed to moderate to severe pollution weather from the preconception period (within 3 months before conception) to the first trimester of pregnancy (conception to 12 weeks)? | ① No ② Yes |
| 11. Have you ever smoked from 3 months prior to pregnancy to recruitment time? | ① No ② Yes |
| 12. Have you been exposed to secondhand smoke from the preconception period (within 3 months before conception) to the first trimester of pregnancy (conception to 12 weeks)? | ① No ② Yes |
| 13. Have you ever drunk from the preconception period (within 3 months before conception) to the first trimester of pregnancy (conception to 12 weeks)? | ① No ② Yes |
| 14. Have you ever taken the following nutrients from the preconception period (within 3 months before conception) to the first trimester of pregnancy (conception to 12 weeks)? | ① No ② Yes |
| 14.1 Vitamin supplementation | 1. No ② Yes |
| 14.2 Folic acid supplementation | 1. No ② Yes |
| 14.3 DHA supplementation | 1. No ② Yes |
| 14.4 Cod-liver oil supplementation | 1. No ② Yes |
| 14.5 Calcium supplementation | 1. No ② Yes |

Table S2. The sample size for the CHD and non-CHD cases at different percentiles.

| Lipid profile | Percentiles | CHD | Non-CHD |
| --- | --- | --- | --- |
| TC | 0-5th | 28 | 1043 |
|  | 5th-35th | 127 | 6300 |
|  | 35th-65th | 118 | 6310 |
|  | 65th-95th | 164 | 6263 |
|  | 95th-100th | 28 | 1044 |
| TG | 0-5th | 27 | 1044 |
|  | 5th-35th | 107 | 6320 |
|  | 35th-65th | 145 | 6283 |
|  | 65th-95th | 150 | 6277 |
|  | 95th-100th | 36 | 1036 |
| LDL | 0-5th | 19 | 1052 |
|  | 5th-35th | 133 | 6294 |
|  | 35th-65th | 104 | 6324 |
|  | 65th-95th | 170 | 6257 |
|  | 95th-100th | 39 | 1033 |
| HDL | 0-5th | 30 | 1041 |
|  | 5th-35th | 152 | 6275 |
|  | 35th-65th | 143 | 6285 |
|  | 65th-95th | 124 | 6303 |
|  | 95th-100th | 16 | 1056 |
| Apo A | 0-5th | 33 | 1038 |
|  | 5th-35th | 145 | 6282 |
|  | 35th-65th | 143 | 6285 |
|  | 65th-95th | 124 | 6303 |
|  | 95th-100th | 20 | 1052 |
| Apo B | 0-5th | 30 | 1041 |
|  | 5th-35th | 115 | 6321 |
|  | 35th-65th | 120 | 6308 |
|  | 65th-95th | 166 | 6261 |
|  | 95th-100th | 34 | 1038 |

Abbreviation: CHD, congenital heart disease; TC, total cholesterol; TG, triglyceride; HDL, high-density lipoprotein; LDL, low-density lipoprotein; Apo A, apolipoprotein A; Apo B, apolipoprotein B

Table S3. Associations between lipid profile in early pregnancy and risk of CHD in offspring after excluding pregnant women with lipid profile levels below the 2.5th percentile and above the 97.5th percentile

|  | unadjusted | model 1 | model 2 |
| --- | --- | --- | --- |
|  | OR (95% CI) *P* | OR (95% CI) *P* | OR (95% CI) *P* |
| TC | 1.140(0.982,1.325)0.086 | 1.136(0.977,1.320)0.097 | 1.110(0.954,1.292)0.176 |
| TG | 1.423(1.135,1.784)0.002 | 1.442(1.145,1.816)0.002 | 1.332(1.051,1.688)0.018 |
| HDL | 0.610(0.424,0.879)0.008 | 0.643(0.446,0.927)0.018 | 0.688(0.475,0.996)0.048 |
| LDL | 1.425(1,186,1.712)0.000 | 1.397(1.162,1.680)0.000 | 1.326(1.098,1.601)0.003 |
| Apo A | 0.571(0.380,0.857)0.007 | 0.640(0.424,0.967)0.034 | 0.567(0.442,1.006)0.054 |
| Apo B | 2.613(1.287,5.306)0.008 | 2.660(1.303,5.431)0.007 | 2.109(1.017,4.371)0.045 |

Abbreviation: CHD, congenital heart disease; OR, odds ratio; CI, confidence interval; TC, total cholesterol; TG, triglyceride; HDL, high-density lipoprotein; LDL, low-density lipoprotein; Apo A, apolipoprotein A; Apo B, apolipoprotein B. Model 1: adjusted for gravidity, assisted reproduction, medication history, expose to air pollution, passive smoking. Model 2: adjusted for model 1+ fasting blood glucose+ BMI

Table S4 Comparison of basic characteristics between included and excluded populations

| Characteristic | Included (n=21425) | Excluded (n=3827) | P |
| --- | --- | --- | --- |
| Age^†^, mean (SD) | 31.9 (4.06) | 32.2 (4.38) | 0.000 |
| BMI (kg/m2) **^‡^** |  |  | 0.431 |
| ＜18.5 | 3074(14.3) | 509(13.30) |  |
| 18.5-<24 | 14671(68.5) | 2614(68.30) |  |
| 24-<28 | 2940(13.7) | 539(14.08) |  |
| ≥28 | 740(3.5) | 136(3.55) |  |
| Missing | 0 | 29(0.76) |  |
| Ethnicity (han)‡ | 20976(97.9) | 3735(97.6) | 0.416 |
| Residence(urban)^‡^ | 19449(90.8) | 3417(89.3) | 0.004 |
| Education level^‡^ |  |  | 0.005 |
| ＜12years | 4842(22.6) | 954(24.9) |  |
| 12-16years | 15085(70.4) | 2599(67.9) |  |
| ＞16years | 1498(7.0) | 270(7.1) |  |
| Missing | 0 | 4(0.1) |  |
| Monthly income, CNY^‡^ |  |  | 0.034 |
| ＜4500 | 9118(42.6) | 1709(44.6) |  |
| 4500-9000 | 8735(40.8) | 1481(38.7) |  |
| ＞9000 | 3513(16.4) | 631(16.5) |  |
| missing | 59(0.3) | 6(0.2) |  |
| Gravidity^‡^ |  |  | 0.000 |
| 0 | 7897(36.9) | 1285(33.6) |  |
| 1 | 1476(6.9) | 271(7.1) |  |
| ≥2 | 12052(56.3) | 2271(59.3) |  |
| Assisted reproduction^‡^ |  |  | 0.004 |
| Yes | 1747(8.1) | 365(9.5) |  |
| No | 19678(91.8) | 3462(90.5) |  |
| Hypertension^‡^ | 3899(18.2) |  | 0.333 |
| Yes | 336(1.6) | 65(1.7) |  |
| No | 21020(98.1) | 3563(93.1) |  |
| Missing | 69(0.3) | 199(5.2) |  |
| Medication history^‡^ | 10387(48.5) | 1859(48.6) | 0.234 |
| Exposure to air pollution^‡^ | 9617(44.9) | 1737(45.4) | 0.512 |
| Smoking^‡^ | 478(2.2) | 92(2.4) | 0.498 |
| Passive smoking^‡^ | 7106(33.2) | 1321(34.5) | 0.073 |
| Drinking^‡^ | 2497(11.7) | 447(11.6) | 0.203 |
| Vitamin supplementation^‡^ | 11229(52.4) | 1994(52.1) | 0.870 |
| Folic acid supplementation^‡^ | 13106(61.2) | 2307(60.3) | 0.436 |
| DHA supplementation^‡^ | 1679(7.8) | 330(8.6) | 0.084 |
| Cod-liver oil supplementation^‡^ | 246(1.1) | 74(1.9) | 0.000 |
| Calcium supplementation^‡^ | 2499(11.7) | 551(14.4) | 0.000 |

Abbreviation: CHD, congenital heart disease; BMI, body mass index.

† Mean (SD), Student’s t -test; ‡ n (%), chi-square test

Figure S1: Directed acyclic graph


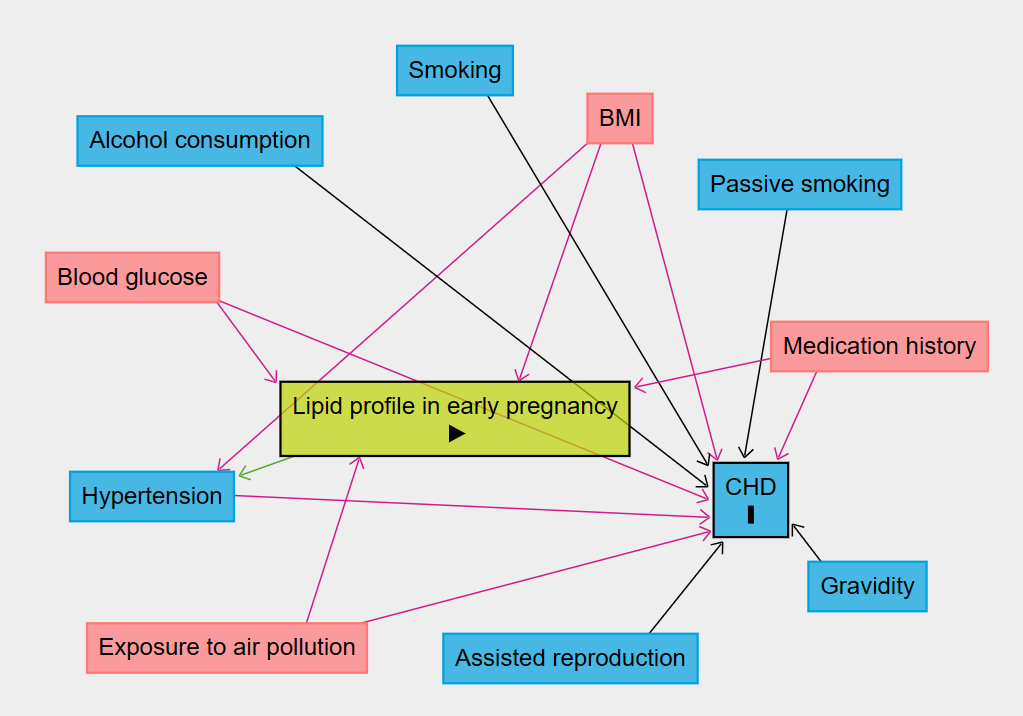


Figure S1: Example of directed acyclic graph (DAG) for the causal effect of lipid profile in early pregnancy on offspring CHD. Variables are represented by nodes on a DAG. Assumed causal relationships between variables are represented by directed edges (i.e., arrows) between nodes. Abbreviation: CHD, congenital heart disease; BMI, body mass index.
